# With an eye on uncertainty: Modelling pupillary responses to environmental volatility

**DOI:** 10.1371/journal.pcbi.1007126

**Published:** 2019-07-05

**Authors:** Peter Vincent, Thomas Parr, David Benrimoh, Karl J Friston

**Affiliations:** Wellcome Trust Centre for Neuroimaging, Institute of Neurology, University College London, London, United Kingdom; Johns Hopkins University, UNITED STATES

## Abstract

Living creatures must accurately infer the nature of their environments. They do this despite being confronted by stochastic and context sensitive contingencies—and so must constantly update their beliefs regarding their uncertainty about what might come next. In this work, we examine how we deal with uncertainty that evolves over time. This prospective uncertainty (or imprecision) is referred to as volatility and has previously been linked to noradrenergic signals that originate in the locus coeruleus. Using pupillary dilatation as a measure of central noradrenergic signalling, we tested the hypothesis that changes in pupil diameter reflect inferences humans make about environmental volatility. To do so, we collected pupillometry data from participants presented with a stream of numbers. We generated these numbers from a process with varying degrees of volatility. By measuring pupillary dilatation in response to these stimuli—and simulating the inferences made by an ideal Bayesian observer of the same stimuli—we demonstrate that humans update their beliefs about environmental contingencies in a Bayes optimal way. We show this by comparing general linear (convolution) models that formalised competing hypotheses about the causes of pupillary changes. We found greater evidence for models that included Bayes optimal estimates of volatility than those without. We additionally explore the interaction between different causes of pupil dilation and suggest a quantitative approach to characterising a person’s prior beliefs about volatility.

## Author summary

Humans are constantly confronted with surprising events. To navigate such a world, we must understand the chances of an unexpected event occurring at any given point in time. We do this by creating a model of the world around us, in which we allow for these unexpected events to occur by holding beliefs about how volatile our environment is. In this work we explore the way in which we update our beliefs, demonstrating that this updating relies on the number of unexpected events in relation to the expected number. We do this by examining the pupil diameter, since—in controlled environments—changes in pupil diameter reflect our response to unexpected observations. Finally, we show that our methodology is appropriate for assessing the individual participant’s prior expectations about the amount of uncertainty in their environment.

## Introduction

The role of the noradrenergic (NA) system in decision making [1] and encoding uncertainty [2] has been explored in great depth, with many studies using pupillary dilation as an index of changes in central adrenergic signalling [3–6]. A central theme of this work is the role of NA in contextualising perceptual inference and planning. This has an interesting connection to the P3 evoked response potential seen in EEG paradigms [7]. The amplitude of the P3b wave increases following presentation of surprising stimuli [8,9] and is thought to signal a change in beliefs about the underlying environmental contingencies [9]–i.e., the updating of context [10]–and might be mediated by NA [1]. However, these accounts of the role of NA in signalling surprise often focus on transient responses following a single unexpected event. Here, we extend this work to show that pupil dilatation tracks a subject’s long-term beliefs (that is, tonic changes to the baseline pupil diameter), spanning multiple aberrant events and how these allow the participant to infer the precision of their environmental dynamics. Precision here refers to the predictability of the next state of the world, given the current state.

Formally, we appeal to the notion of a generative model. This is central to theoretical treatments of the Bayesian brain and, more generally, active inference. These accounts frame brain function as a process of inference that depends upon an internal generative (predictive) model comprising prior beliefs about variables in the world, and (likelihood) beliefs about how these give rise to sensory data. On observing sensory data, creatures can use their internal model to update posterior beliefs about their environments. Posterior beliefs can then be used to compute empirical prior beliefs about the future (i.e., planning as inference), using temporal contingencies in the generative model. Our focus here is how the brain handles uncertainty about these contingencies.

The encoding of uncertainty is essential in enabling animals to predict confidently (or not) what might happen next [11]. From the perspective of the Bayesian brain, this is the process of using beliefs about the past to form (empirical) prior beliefs about the present. Crucially, the confidence (precision) in these priors determines the relative weighting of prior and sensory influences on perceptual or state inference. This has relevance for the role of abnormal prior beliefs in pathology, where under-confident priors fail to contextualise inferences drawn from sensory data or where excessively confident priors support false inferences in the presence of contradictory sensory data [12]. Of particular relevance to this work are those conditions that have been associated with abnormal NA signalling—for example, autism [13,14].

We start by introducing a few technical concepts. Following this, we describe our experimental design and data collection. With these data, we test the hypothesis that the pupil diameter closely tracks the precision inferred by the participant in a volatile setting. We build on this formulation to propose a technique that quantifies prior beliefs, which could be used in a clinical context to phenotype individuals, in terms of their prior beliefs about precision and volatility (e.g., that might underwrite autistic symptoms).

### Precision, inference and Markov decision processes

Adaptive engagement with the world requires an understanding of our sensations in terms of the latent (hidden or unobservable) states that generated them. This requires an internal (generative) model of the world that can be used to make predictions about sensory input [15]. These generative models are necessarily complicated (i.e., usually deep, dynamical and nonlinear), to capture the subtleties of our (deep, dynamical and nonlinear) environment. Despite the complexity of such models, they can be constructed by combining relatively simple models [16]. The simplest that accounts for perceptual inference and planning—in a changing environment—is a Markov decision process (MDP) [17]. Technically, in this paper we use a hidden Markov model (as we do not model any decisions), but we retain the MDP rhetoric to emphasise that these results generalise to situations that require active sensing of the world [17,18]. In the following section we provide a brief outline of the inversion of this type of generative model. Readers familiar with this sort of modelling are invited to skip this section.

**Fig 1:**
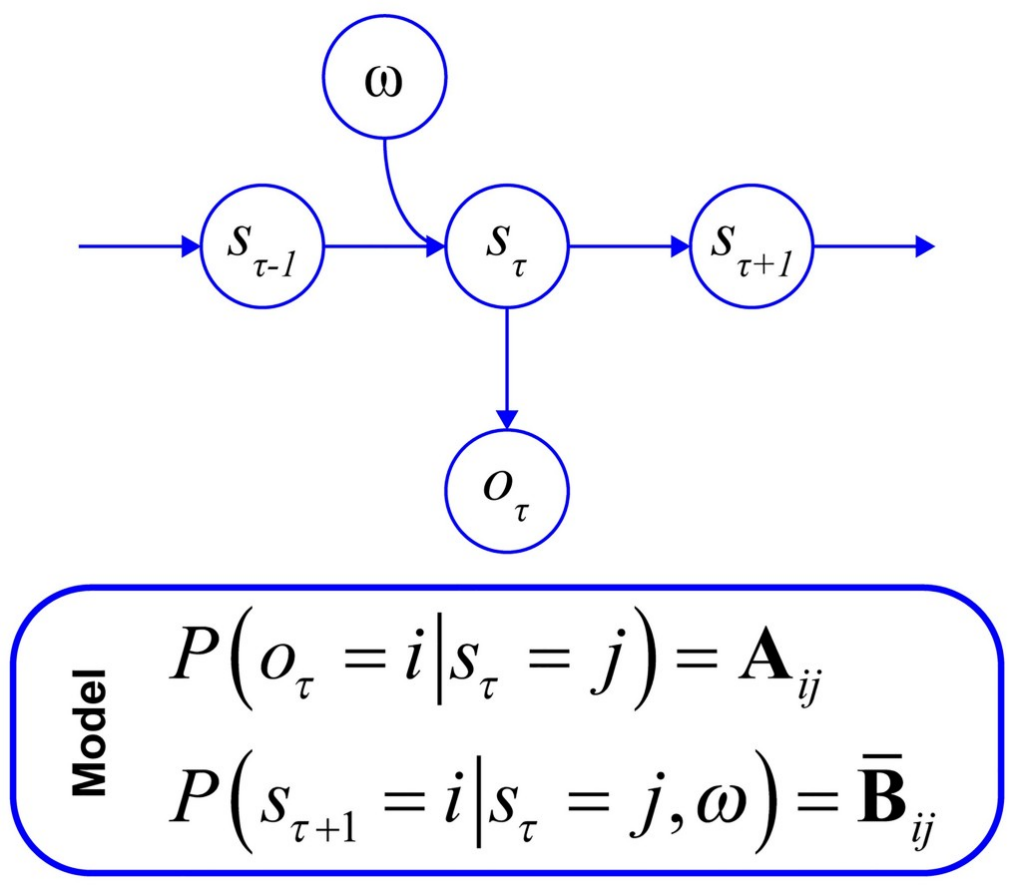
A (simplified) Bayes net. This is a form of graphical notation that expresses the conditional dependencies in the generative model. Random variables are shown in circles, with arrows indicating causal influences. The progression from one state (*s*_*τ*_) to the next (*s*_*τ*+1_) is affected by the precision (*ω*) of the transition matrix. This stochasticity results in randomness in the observed outcomes (*o*_*τ*_). The panel below specifies the parameterisation of this model. Notably, in addition to (likelihood) beliefs about how states generate data (**A**), and beliefs about state transitions (**B**), we need prior beliefs about the precision of these transitions. These take the form of a gamma distribution, *P*(*ω*) ∝ *βe*^−*β*·*ω*^, where the current prior belief (*ω*) is a function of the most recent posterior beliefs (***β***). This has the convenient property that the expectation of the prior beliefs as we update them are the value of the most recent posterior beliefs [22].

An MDP treats the world as comprising a series of states (*s*) that are hidden from an observer. The transitions among these states over time represent the (stochastic) dynamics of the environment, and are defined by a (square) transition matrix that we denote by **B** (see Fig 1 for a Bayes net representation of this process). These states give rise to observable outcomes that act as the observer’s sensory stimuli. The relationship between the hidden states and the outcomes they generate is expressed as a likelihood matrix, **A**. These probability distributions are not trivial: to motivate their importance we appeal to the Good Regulator Theorem [19]. This theorem says that ‘every good regulator of a system must be a model of that system.’ From this, one may intuit that if a creature inhabits, and wishes to interact with, an environment defined by stochastic state transitions, this creature must be able to estimate the precision (i.e., the negative entropy) of these transition densities. The ensuing inference about environmental dynamics is intertwined with beliefs regarding the likelihood mapping from states to outcomes, since it is only these outcomes that an agent can observe [17]. If the dynamics of the environment are deterministic, and state-to-outcome mappings are well understood, the agent is likely to have precise beliefs about the nature of its environment, and is therefore able to accurately predict what it may expect to see in the future [20]. However, when these mappings from states to outcomes are not deterministic and where state transitions are themselves stochastic—the agent is presented with a confound, since poor inferences about the nature of state-to-outcome mappings may have a detrimental effect on inferences about state transitions [20,21].

**Fig 2:**
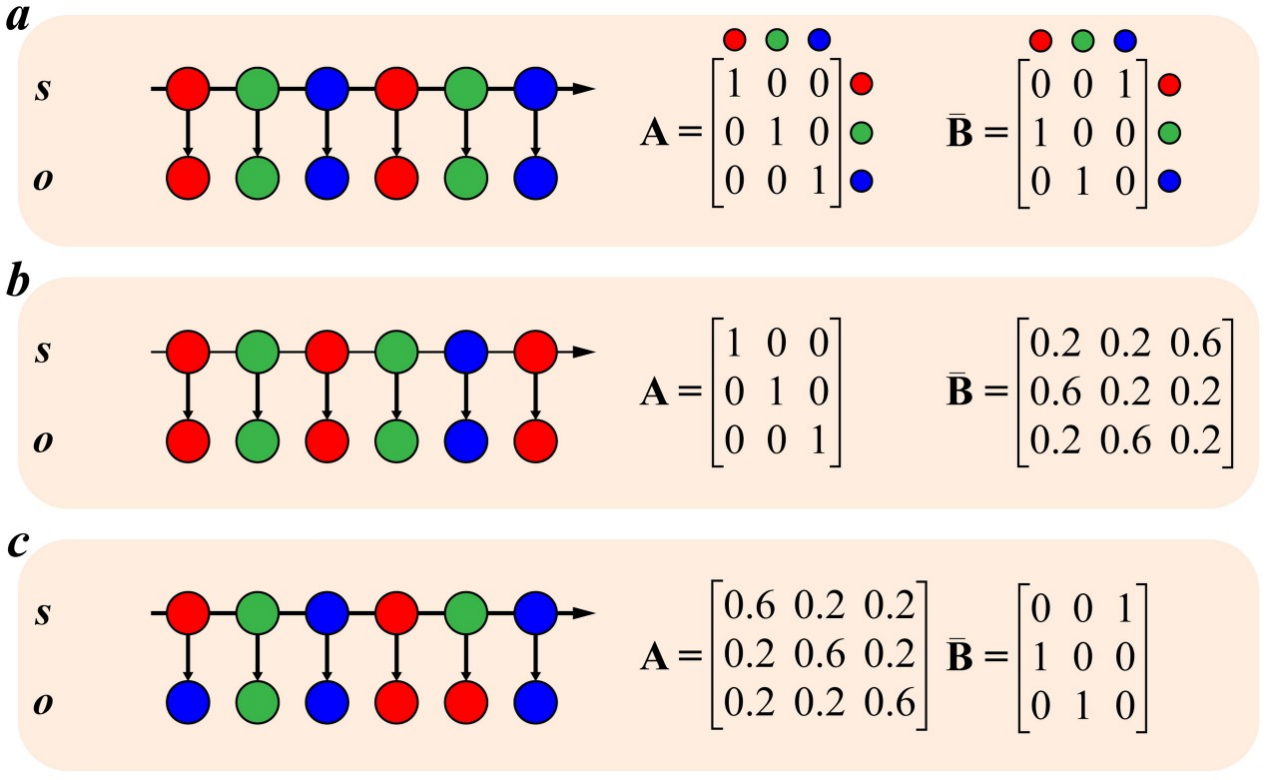
Sequences of outcomes emerging from random transition dynamics. Fig 2a shows the sequence of outcomes (*o*) produced by the sequence of states (*s*) when the transition matrices for both sequences are deterministic. Fig 2b shows the sequence of states, and outcomes those states generate, when the matrix defining the transitions between states (**B**) is not deterministic. Even though the transitions from states to outcomes are still deterministic, as in Fig 2a, the final sequence of outcomes is different. In Fig 2c we show the consequences of introducing randomness into the transitions (matrix **A**) from states to events. The sequence of states remains the same as in Fig 2a, but the outcomes generated by each state are random, resulting in a different sequence of outcomes. Figs 2a and 2c demonstrate how randomness in the transition matrices can results in unpredictable sequences of outcomes.

We represent these imprecise state-to-outcome mappings and state-to-state transitions in the **A** and **B** matrices, respectively (Fig 2). Imprecise state transitions (a non-deterministic **B** matrix) define a volatile environment. In other words, volatility is equivalent to the inverse precision of the **B** matrix. In a volatile world, even if an animal accurately infers the likelihood mappings and state transitions, the stochastic nature of these dynamics means the agent’s beliefs about what will happen next are necessarily imprecise [22]. Imprecise beliefs over state transitions leave the animal with no way of predicting what might come next. This has been referred to as ‘unexpected uncertainty’, in contrast to ‘expected uncertainty’ (that maps to imprecision of **A**) [2,6].

### Volatility and pupil dilation

Previous work has suggested that acetylcholine (ACh) and noradrenaline (NA) act as the neurochemical analogues of the precision over state-outcome mappings (i.e. the **A** matrix) and the precision over state transitions (defining the empirical prior and **B** matrix) [2] respectively. In other words, ACh is thought to be involved in signalling confidence in our beliefs about the likelihood of what we might see, in a given state, while NA moderates our confidence in prior beliefs about the state we may find ourselves in next [2,3,5,23,24]. In this work, we use these proposed relationships—between central noradrenergic signalling and volatility signalling—to motivate predictions about changes in pupil diameter based on environmental volatility. Given that circuits in the brain—that update beliefs over the likelihood mappings from states to outcomes—are thought to use ACh as a transmitter [2,23,25–29], a potential confound arises: pupil diameter, particularly the tonic changes examined in this work, may also depend on beliefs about the likelihood of certain stimuli. We therefore consider the possibility that beliefs about sensory mappings may confound the effects of beliefs about state transitions on the pupillary response; acknowledging that beliefs about the likelihood might also vicariously influence beliefs about transitions. Put simply, an unexpected observation could plausibly be explained by imprecision in either the **A** or the **B**-matrix.

### Parameterising volatility

To optimise beliefs about environmental uncertainty (i.e., precisions), we must first specify how precisions are parameterised. To pursue this formally, we express the precisions as inverse temperature parameters, such that the precision of state transitions is given by ω. This adjusts a (source) transition **B** matrix by virtue of a Gibbs measure (i.e., a softmax function), as shown in Eq 1. Here, precision is an exponent on the elements of the transition matrix, which is then normalised, to produce the agent’s beliefs about state transitions [22].

B-τ,ij=Bijω∑kBkjω (1)

**Fig 3:**
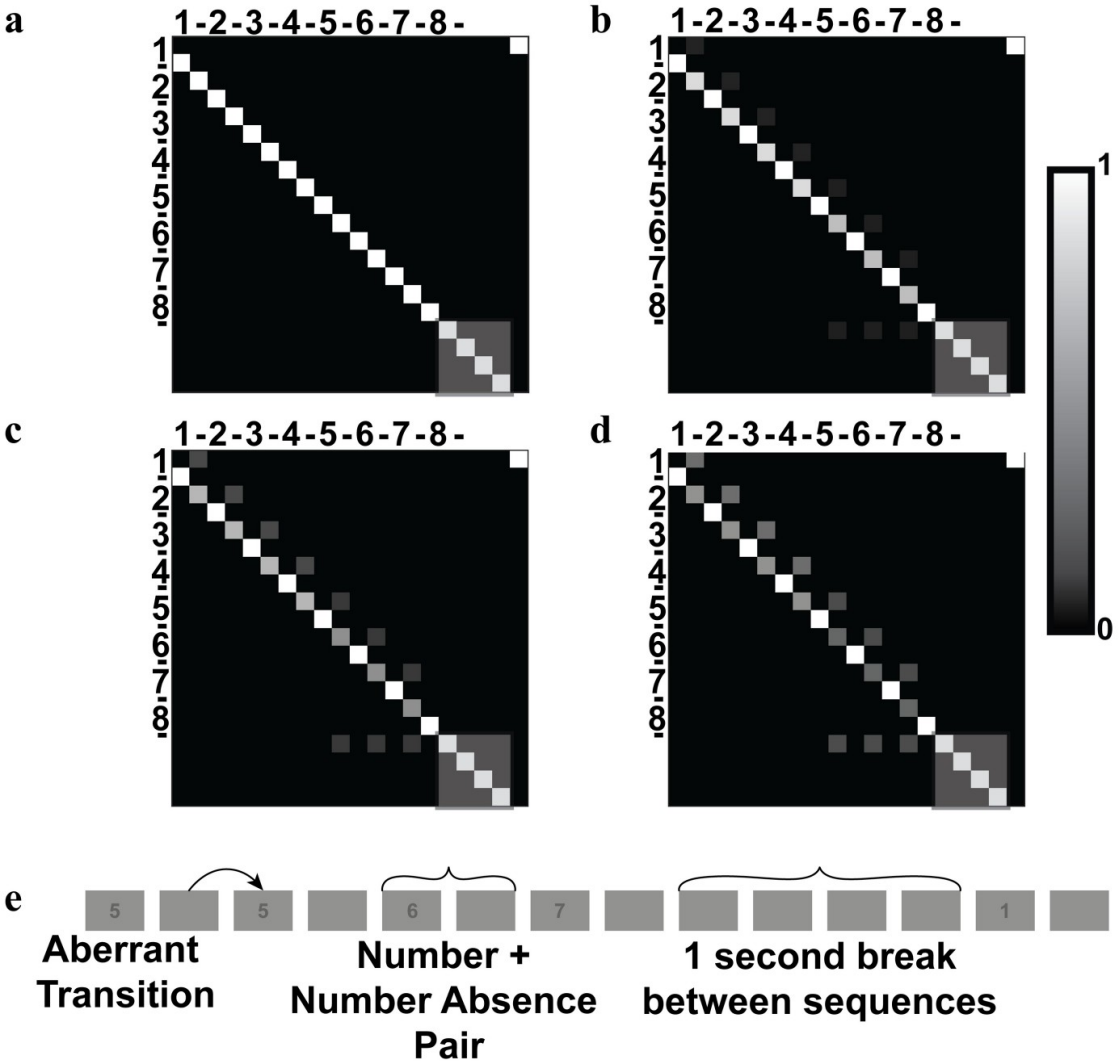
B matrices created for the generative process. Lighter values indicate probabilities closer to 1. As the precision decreases, shown in Figs 3a through 3d, the probability of a number-absence state transitioning to the next number decreases, while the probability of a transition to the preceding number increases. The grey transparent box covers transitions into and within the final 1s break. Separate states are required for each of the number-absence states to ensure this matrix generates a reliable sequence of numbers. Number-absence states following 5,6,7 may transition to the first of the block of 4 number-absence states as the precision decreases, to ensure that the sequence of numbers is always 8 long. These matrices can result in unexpected sequences, as shown in Fig 3e, which is drawn from the matrix defining a scenario with precision = 0.39, since it only has one error.

Eq 1 describes how transition matrices are generated from a source matrix. This produces the transition matrices shown in Fig 3, with all 4 derived from the same ‘source’ matrix. Intuitively, a high prior precision reflects high confidence in prior beliefs, and would be represented by a large value of ω. If ω were to equal infinity, this would represent absolute confidence, and results in a purely deterministic transition matrix, as is shown in Fig 3a. Smaller values of ω (Fig 3b–3d) represent increasingly less precise beliefs, resulting in increasingly stochastic transition matrices, and a greater propensity to accommodate randomness in the environment.

The updated, normalised (denoted by the bar notation), **B** matrix is then used to update the expected precision given new sensory outcomes. Under ideal Bayesian observer assumptions [22] this update can be cast as a gradient ascent on variational free energy (a lower bound on log model evidence). Specifically, this scheme updates the volatility (inverse precision, β = ω^−1^) using the sum of prediction errors, weighted over all possible transitions, as shown in Eq 2. These prediction errors represent the difference between the observed state transition and the expected state transition, where the expected transition is calculated using the updated **B** matrix calculated in Eq 1. The ensuing error term is shown in Eq 3.

$$\dot{\beta }=\beta -\sum_{\tau }\text{ln}\left(B_{\tau }\right)∙\varepsilon_{\tau }-\beta$$ (2)

$$\varepsilon_{i,\tau }=(s_{\tau +1}-{\overset{-}{B}}_{\tau ,i})s_{\tau }$$ (3)

$$s_{\tau }=\sigma ({\text{v}}_{\tau });\;{\dot{\text{v}}}_{\tau }=\omega (\text{ln}(B_{\tau -1}\cdot {\text{s}}_{\tau })+\text{ln}(B_{\tau }∙s_{\tau +1})+\text{ln}\left(A∙o_{\tau }\right)-\text{ln}\left(s_{\tau }\right)$$ (4)

Eqs 2 and 3 show how the inferred volatility is inextricably linked to violations of expected transitions, as inferred by the subject [22]. Here, σ refers to the softmax function. Importantly, the non-italic β in Eq 2 represents the prior beliefs an agent has regarding the volatility of the environment (so β^-1^ = ω are the prior beliefs over precision). Eq 2 therefore shows how the agent’s posterior beliefs about the current environmental volatility (*β*) depends on their prior beliefs. The formulation in Eq 3 provides a useful intuition on belief updating for volatility or precision. It says that, for every possible current state, we compute the difference between the expected next state and the posterior beliefs about that state. These errors are weighted by the posterior probability of the current state. Larger errors then induce greater updates in beliefs about the volatility. Framing belief updating in terms of state prediction errors connects this aspect of active inference to recent work suggesting that much of noradrenergic phenomenology can be reproduced by appealing to similar error terms [30].

Eq 4 shows how we estimate posterior beliefs about the states, and the influence of the volatility on this belief updating [22]. This shows that, in a highly volatile world (low ω), the influence of beliefs about the future and past should have little influence over beliefs about the present, and we should rely to a greater extent upon sensory evidence. In contrast, in minimally volatile environments, we should depend more upon empirical priors from the past and future. When inferring state trajectories, we can use these posterior beliefs to evaluate Eq 3 and update beliefs about volatility.

There is a large literature on modelling of volatility in dynamical systems that rely upon autoregressive or Kalman filter like models. While important for cognitive sciences and psychology [31,32], these also find application in the domain of financial modelling and economics [33,34]. Some of these approaches rely upon the use of stochastic differential equations for continuous variables (or their associated density dynamics [35]), while others rely upon probability transition matrices. In the former, volatility is simply the variance of random fluctuations, while in the latter it takes the form of a temperature parameter. Common to all, is the notion that the current value of a latent variable does not deterministically predict the next value. All explicitly or implicitly appeal to the imprecision of predictions about the next state, given the current state, as a measure of volatility.

Previous work has considered the updating of precisions in continuous state space models, using a hierarchical gaussian filter [36]. In this scheme, beliefs are held at multiple hierarchical levels, with belief updating driven by prediction errors. The precisions at each level are dynamic, and encode the uncertainty (or the volatility) about fluctuating continuous states of the environment [36]. Other approaches have considered a delta-rule style belief updating, which has been combined with Bayesian approaches to form a Bayesian delta rule [37,38]. These formulations have previously been used to examine the relationship between noradrenergic signalling and the estimation of volatility in both a neurotypical setting, with and without reward, [6,38] and in the case of patients with autism [14]. Indeed, optimising beliefs about the uncertainty of state transitions is an essential feature of cognitive flexibility, allowing us to anticipate changes in task contingencies. This means we can assess the relevance of recent events in predicting what might come next. This regulation of beliefs is synonymous with the learning rate in reinforcement learning [6,24,38,39].

In this work we focus on the uncertainty about the environmental contingencies. By formalising the Bayesian updating thought to occur in the brain [6,22,40], we can quantify the prior precision (i.e., confidence) participants afford their beliefs about environmental volatility by examining the effect on the belief-updating when presented with a unpredictable outcomes [22]. Crucially, our model makes predictions about the online encoding of uncertainty and accompanying pupillometric responses. This allows us to examine the tonic responses of the pupil without using summary statistics, as in previous work [6,41]: usually, trial-to-trial fluctuations in the pupil diameter are measured by taking the average dilation or the change relative to baseline. In this work, we generalise this examination of the trial-to-trial fluctuations in pupil diameter by explicitly parametrising it as a function of inferred precision [22]. This allows us to quantify a participant’s prior precisions over environmental volatility based upon observable (pupillometry) responses—a capability that holds promise for applications in theoretical, computational and clinical neuroscience [14,22,42].

## Materials and methods

To test the hypothesis that pupillary responses are, in part, mediated by the encoding of uncertainty, we assessed the evidence for alternative models of pupillary dilatation afforded by pupillometry data from 9 healthy participants. We generated plausible models to account for pupillary responses, considering optical factors, our formulation of the inferred environmental volatility, and possible interactions between these factors (i.e. how beliefs about environmental volatility moderate the pupillary response to luminescence). In this section, we explain the design and rationale of the stimuli, the pupillometry data collection protocol, stimulus generation and model specification.

### Ethics statement

The study was approved by the UCL Research Ethics Committee (Project ID Number 4356/002). Both oral and written informed consent was obtained from all participants.

### Pupillometry data collection

We recruited 9 participants between the ages of 18–35 with no reported psychiatric history and neurotypical development. All participants’ data are included in the analysis, and all participants completed the 16 blocks. Each block lasted for just over 2 minutes; allowing for short breaks between blocks (to avoid discomfort). Each session lasted for around 1 hour. Participants rested their head in a chinrest 0.5m away from a stimulus presentation screen. Dark numbers on a grey background were used to reduce the effect of pupillary dilatation in response to changes in illumination [37], and the lights were dimmed (high illumination leads to a constricted pupil and restricted dilatation). Quiet was maintained during each experiment to avoid dilation in response to auditory cues [43], which would effect the level of surprise [6]. Pupil area was monitored in the left eye using an EyeLink 1000 desktop mount (SR Research, sampling rate: 1000Hz), with calibration performed before the first experiment, as well as after any periods where the participant moved from the chin rest.

While fluctuations in pupil diameter can be attributed to a luminance effect, due to the presentation of numbers on the screen, we note that this pupil dilation could alternatively (and perhaps also) be due to increased attention [44]. This effect has been reported during auditory paradigms: a recent study showed that the conscious processing of regularities in an auditory paradigm induces a pupillary dilation [4,45,46].

### Task procedure and stimulus generation

**Fig 4:**
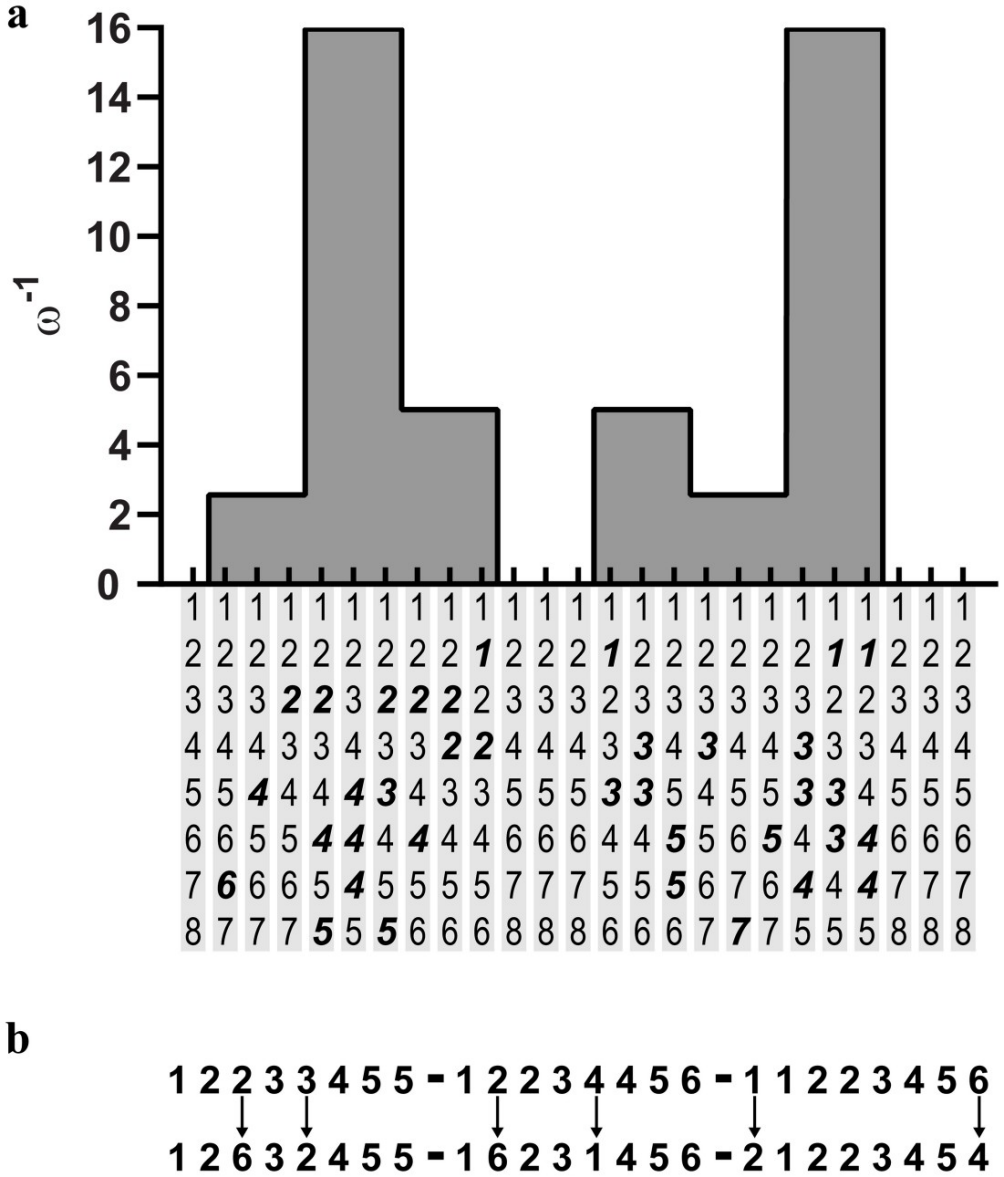
Sequences and their volatility. 25 sequences are presented in each block. The first sequence is 1–8. The following 24 sequences are placed into 8 sets; each set has a predetermined volatility; alternatively, the y-axis gives the volatility of the generative process. The exact sequences are shown hanging below the x-axis in Fig 4a, where unexpected numbers resulting from an aberrant transition are highlighted in bold font. ω^-1^ is used here to distinguish the volatility of the generative process from the participants’ inferred volatility β. Fig 4b shows the composition of combination sequences. The same set of 25 sequences is taken, in the same order, to generate the combination sequences. Two numbers in each sequence are exchanged for different numbers to generate a new sequence. The upper row shows sequences with varying degrees of volatility (but precise likelihood probabilities), while the lower row shows these same sequences augmented as if they were generated from imprecise likelihood distributions.

To measure the pupillary response to changes in environmental volatility, we presented sequences of numbers increasing from 1 to 8. These were generated using a probability transition matrix (details below), analogous to that used in the generative model (see Fig 2). We chose numbers because prior exposure to number sequences means that participants are ‘over-trained’, and do not need to learn new sequences. To increase the volatility of this stimulus, we decreased the precision of the transition matrix used to generate the sequence. This introduced violations of the 1–8 sequence. Every sequence, irrespective of the number of violations, comprised 8 numbers. Each number was presented for 250ms, followed by a 250ms with no stimulus. In other words, each number was always followed by the absence of a number, such that each of these pairs lasted for 500ms. After 8 pairs, there was a delay for 1 second. The small 250ms spaces between numbers were required to make transitions distinct (for example, without these interludes a transition from a 2 back to a 2 would simply look like an extended presentation of the number 2). The 1 second breaks between sequences helped prevent transitions such as 8 to 1 as one sequence ends and another began immediately after—as this could equally well be seen as a an unexpected transition. Numbers were presented in a dark font on a grey screen; a truncated example of a sequence is shown in Fig 4e.

To account for the 250ms number absences between the numbers, and a final 1s break between states (comprising 4 consecutive 250ms number absences), we required a 20x20 probability transition matrix. We ensured that all numbers invariably transitioned to a number absence state, but number absence states could stochastically transition back to the previous number, or to the next number. Specifically, we specified the probability of the ‘correct’ transition (i.e. to the next number numerically) as 0.99, and then split the remaining probability mass among the remaining possible state transitions. We then selected 4 levels of volatility that would give, on average, 0, 1, 2 or 3 aberrant transitions within a sequence of 8 numbers. These volatilities were used to create the matrices shown in Fig 3a–3d. To generate the sequences shown in Fig 4, we iterated using the respective matrix 20 times, and—if the resultant sequence was suitable (i.e. composed of 8 numbers, to ensure all sequences are the same length)–the sequence was accepted; otherwise we generated a new sequence.

A single block comprised 25 sequences in total and each participant performed 16 blocks. The first of these sequences was simply 1–8. The remaining 24 sequences were divided into 8 sets of 3 sequences. Each set was assigned a level of precision, and the appropriate **B** matrix was used to generate each of the 3 sequences. The precisions of the sequences throughout the experiment, and the sequences themselves, are shown in Fig 4. These sequences and their ordering were kept constant throughout all 16 blocks. To ensure participants maintained focus, we asked them to perform an incidental task: they were asked to tap on a tap-counter every time they saw a specific number (this number was different in each block). They were explicitly asked not to count how many times they saw the target number, but rather to focus on the next number (which they were told should always be 1 greater than the number they just saw).

Above, we noted the possibility of an interaction between beliefs about likelihood mappings and state transitions. In the context of these sequences, we looked for this interaction by creating combination sequences for 8 of the 16 blocks that participants completed. In these sequences, we took the basic sequences given in Fig 4, randomly selected 2 numbers and switched them for different numbers. Examples of these switches and the resultant sequences are given in Fig 4.

In summary, each participant completed 16 blocks. 8 are composed of the basic sequences detailed in Fig 4, while 8 are combination sequences, constructed in the manner shown in Fig 4. Importantly, in each set of 8 blocks, the sequence of numbers was exactly the same. In a post-hoc debrief, participants were asked what they noticed about the sequences. None reported that the sequences were the same (either within the sets of 8 blocks or between the sets of 8 blocks), and all commented that they simply ignored the random numbers in the combination sequences, which has a heuristic similarity to the results shown by Parr and Friston (random stimuli that have no informative value are down sampled/ignored) [47]. Following pre-processing (detailed in the next section), we compared the time series generated from the basic sequences to those generated by the combination sequences. We selected random sections of the time series and used their mean and variance to look for a statistically significant difference in the time series and found none. We ran all analyses (see sections below) on both data sets separately, yielding similar results (though with somewhat larger error due to increased noise from reducing the sample size—see 9a insert for an example of these results). We were therefore able to pool the blocks, and do not make any further distinction between the basic and combination sequences.

### Pupillometry data analysis

Following acquisition, all data were processed with the same protocols, which are well established in the literature. First, blinks were removed by identifying data for which the pupil diameter is 0. These time points are padded by 150ms either side, removed, then replaced by linear interpolation [6,48]. We then regressed out the effect of a temporal drift, the presence of a violation (of both types in the combination series) and the presence of a target number (those requested for the counting task) [6,49]. The data were then mean centered, low-pass filtered below 10Hz, and down-sampled to 10Hz. Since the analysis we performed later was in the time domain, we had no need to respect the Nyquist frequency. Down sampling to 10Hz from 1000Hz was required since the predictions we generated (see next section) were generated at 4Hz.

**Fig 5:**
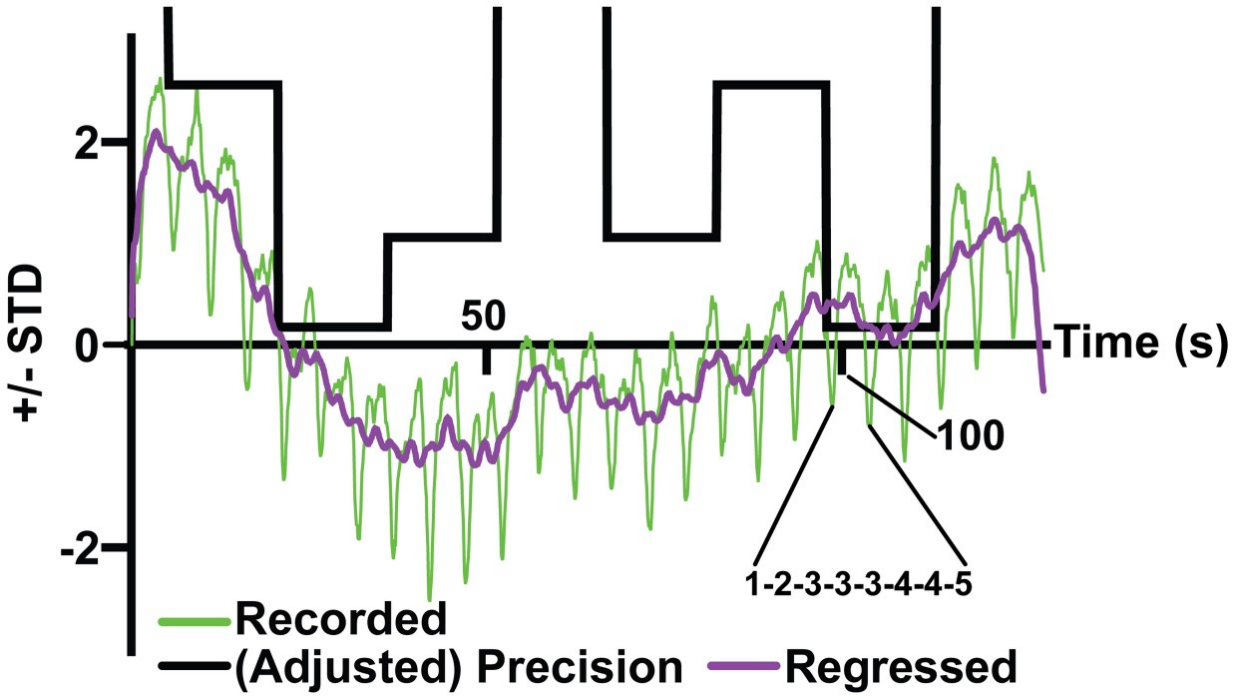
Grand mean of data. This figure shows the data when averaged across all blocks and participants. There are 25 oscillations, which correspond to the 25 sequences, and we explain this in terms of an optical effect. In the next section, we propose that the slow fluctuations that characterise this data are explained best by inferred precision. The grand mean data is shown in green, while the data with the fluctuations regressed out is shown in purple. A trace indicating the precision of the sequences is overlaid—referred to as the adjusted precision, to differentiate from the inferred precision of the participants.

Finally, the data were normalised by their standard deviation, such that the final time series represents the number of standard deviations from the mean diameter. This ensured that we could average the data over subjects, while allowing for the fact that some participants’ responses may have overall smaller pupillary responses due to differential sensitivity to the luminance of the screen. At this point, the data from all 16 blocks for a given participant were averaged together; such that our data-space now comprises 9 time-series (one for each participant) performing exactly the same tasks. This allowed us to construct an ‘event related average’, analogous to the approach used to find evoked responses in EEG research [50]. The grand mean of this average, over subjects, is shown in Fig 5.

### Simulations

**Fig 6:**
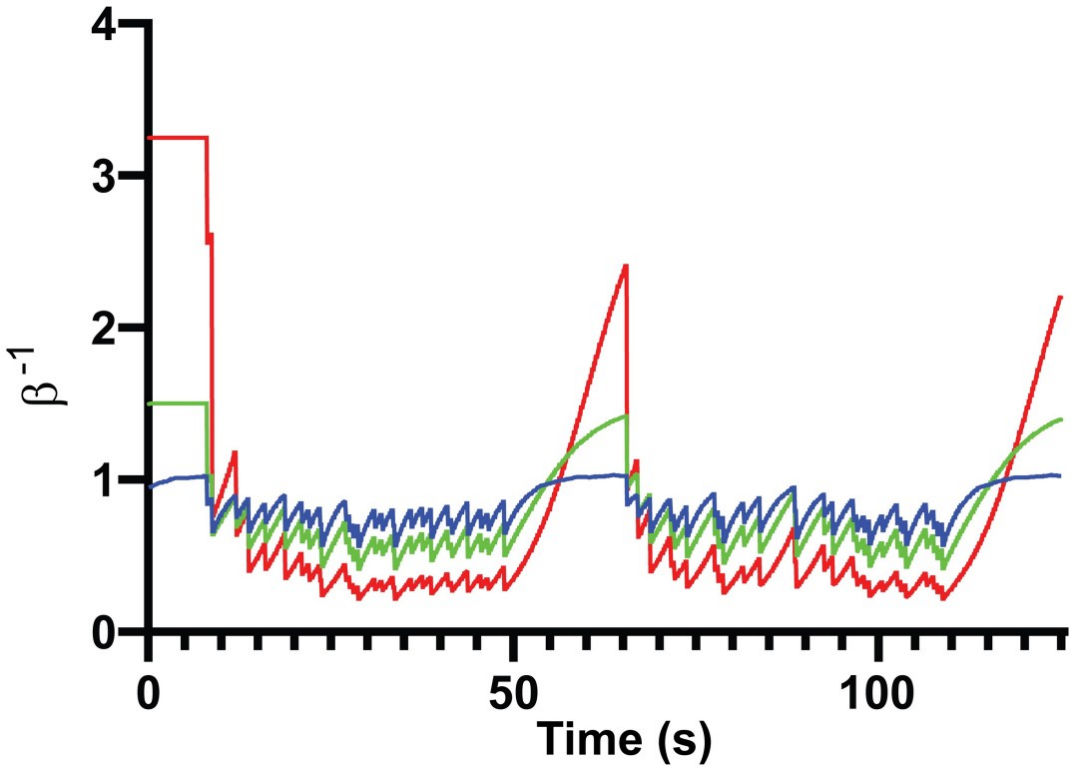
Simulations of the inferred precision. Three simulations are generated with prior precisions of 1, 1.5 and 3.25, in blue, green and red respectively (see Eq 2 for a formal definition of prior precision).

To test our hypothesis (that the central adrenergic system mediates Bayes optimal updating of beliefs over volatility), we simulated belief updating in response to our stimuli. The simulations were performed by iterating Eqs 1, 2, 3 and 4 in a Matlab script customised from spm_MDP_VB_X.m (details in supporting materials). This scheme inverts a generative model based on an MDP to provide free energy minimizing solutions to the underlying active inference problem (that entails the solution to Eq 2). Inference about precision is assumed to proceed over a longer time-scale than state inference (inference about the precision requires beliefs about the current state, which must be inferred from the observed outcomes over a number of time-steps). From Eq 2 it is clear that the inferred volatility is dependent on the prior beliefs over precision (β^-1^). We therefore generated simulations of ω with a range of β^-1^ from 0.3–20. The inverse of the ensuing precision is then taken to be the inferred volatility. Different prior precisions have a profound effect on the shape and scale of belief updating, as can be seen in Fig 6. These simulations are generated at a 4Hz frequency (notice this is the frequency of the stimulus), and we therefore need to up-sample this to 10Hz (by linear interpolation) for comparison with our empirical data. While it may appear as if the time-course in Figs 5 and 6 depends only upon whether numerical sequences are violated, it is actually a little subtler than this. The nature of the response is highly dependent on the prior precision of the participant and the participant’s current inferences about precision. Furthermore, in the short periods of differing volatility that our experiment affords, participants with particularly high beliefs over the environmental volatility are less likely to track these small changes, since they are already expected, whereas participants with strong beliefs over the precision of the environment experience a greater prediction error and will track these changes. Notably, even as the pupil responds to individual aberrations that allow updating of beliefs about precision, the tonic state will change to reflect these beliefs.

### Model space

To test for the hypothesized effects of the inferred precision on pupil diameter, we took the grand mean of the data to generate a pupillometry time series, averaged over all blocks for all participants. Noting the shape of the data (Fig 5), we wanted to consider the balance between optical effects (changes in luminance due to the numbers presenting on the screen) and the updating of inferred environmental precision. With this in mind, we generated 4 plausible models, summarised in Table 1. The explanatory variables detailed in this table accommodate the photic stimulation (effect of numbers on the screen) in each model, and then build on this to consider more comprehensive models of pupillary responses. Model 1 comprises photic stimulation only. This represents a null model. Model 2 contains the photic stimulation and an interaction effect, where the inferred precision acts in concert (i.e. non-additively) with the optical effects. Model 3 contains the photic stimulation and the inferred precision, suggesting that the inferred precision acts independently from optical effects to drive pupillary dilation. Finally, model 4 contains all three effects (optical, interaction and precision). These models are summarised in Table 1. We included a further two interesting models: Model 5 supposes that there are no tonic effects beyond a slow return to baseline following an unexpected event (note this supposes that the tonic effects are simply a due to slow dynamics of the pupil in response the phasic effects). Model 6 supposes that the participant immediately knows the current environmental volatility, rather than having to infer it from the observed data.

**Table 1:** Model designs.

**Model 1** Photic Stimulation	**Model 2** Photic Stimulation Interaction Effects
**Model 3** Photic Stimulation Inferred Precision	**Model 4** Photic Stimulation Interaction Effects Inferred Precision

### Model fitting

Taking inspiration from the field of neuroimaging, we analysed the pupillometry data using a general linear convolution model [44,49,51,52], comparing the evidence for each model using Bayesian general linear regression [53]. The prior expectation of regression parameters were set to 0 within uninformative prior variance. The results presented below were robust to changes in this prior variance. This Bayesian general linear model (GLM) allows us to balance the increase in accuracy from additional regressors in models 1–6 with the accompanying increase in complexity. We convolved our stimuli with 5 gamma functions (with associated parameters), which can be reasonably expected to model the pupillary response to our stimuli—in the spirit of a pupillary response function; i.e., the pupillary response to neuronal afference (modelled by inferred precision). While 5 gamma functions are not required to model pupillary dilation (indeed, analysis of the parameters of each gamma function suggest only the widest gamma function is necessary), we did not want to make any prior assumptions about the pupillary response function, and therefore began with a range of possible functions. We retain this full range to allow for the simulations shown in the results section.

**Fig 7:**
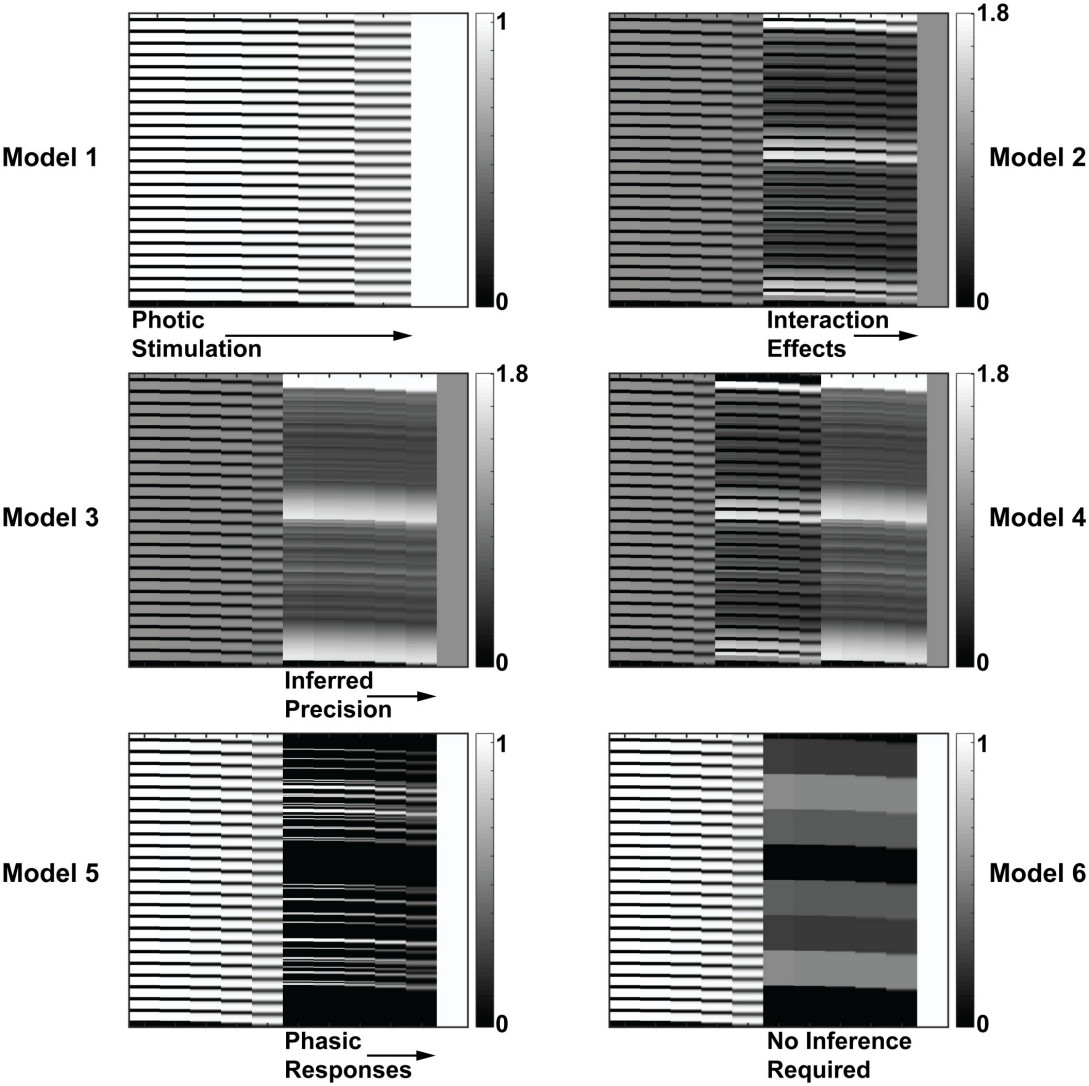
Example design matrices. Design matrices for models 1–6, corresponding to the stimuli detailed in Table 1 and the additional two naïve models (models 5 and 6). Each column indicates a single regressor, while each row is a point in time. In each model the final column (un-numbered) is a constant factor that accounts for the z-scoring performed in the pre-processing. The scale bar on the right indicates the values of the cells in the matrix.

Photic stimulation was modelled as a boxcar function encoding the presence of a stimulus, the interaction term is the mean centred product of the simulated precision and the optical effects, while the precision terms are generated as described above. These explanatory variables are then convolved with a basis set comprising (five) gamma functions, such that the design matrix for model 3 has 10 columns. We added a constant term to account for the z-scoring performed in the pre-processing. Examples of these design matrices for a β^−1^ of 1.75 are shown in Fig 7. Model comparisons are performed with flat priors over each model, to avoid favouring one model over another.

## Results

**Fig 8:**
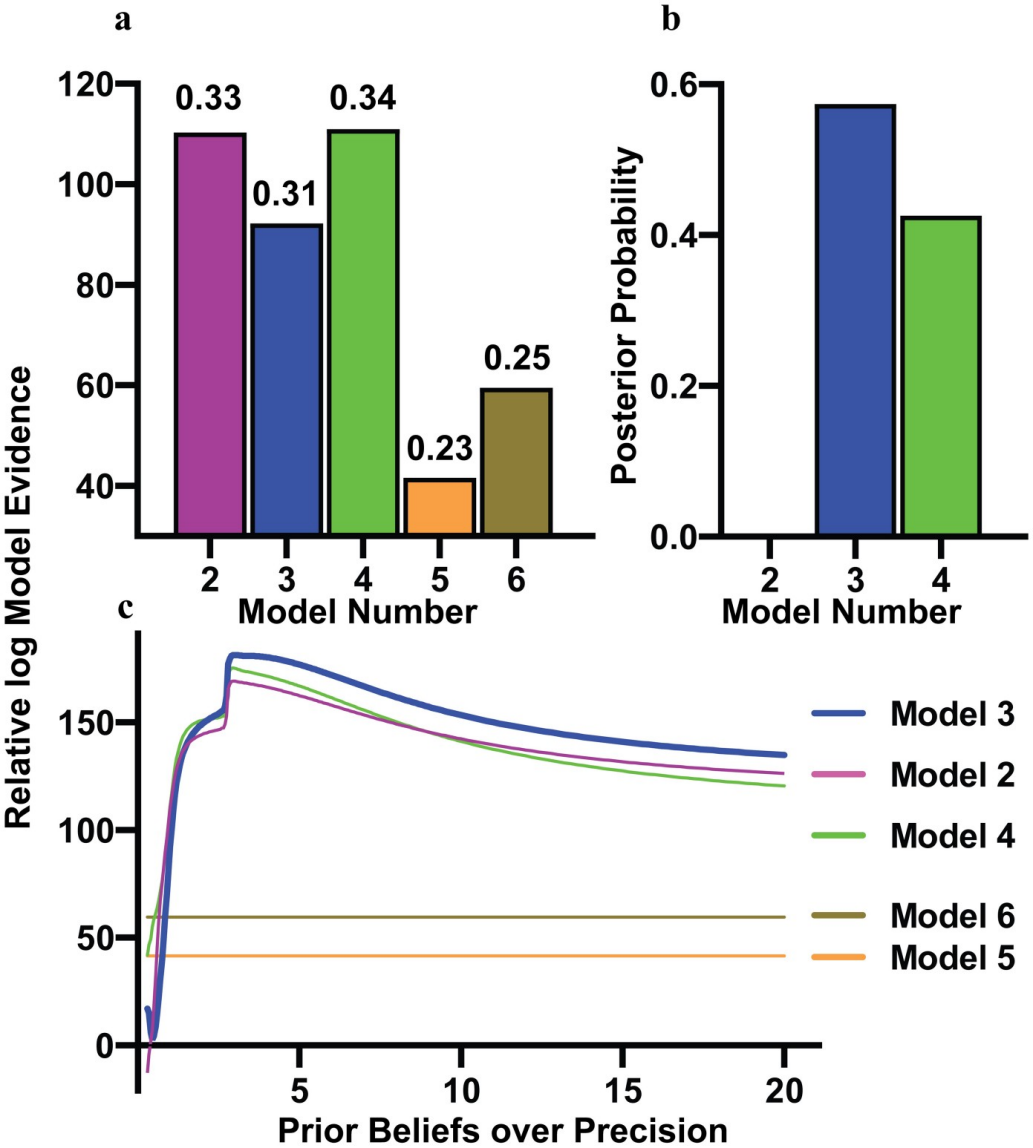
Bayesian comparison of alternative models relative to the null model. Fig 8a shows a bar plot of the log model evidence relative to that of the null model at a prior precision of 1, with the R^2^ scores for each fit provided above each bar (the lowest of our candidates, see Fig 9a). Fig 8b shows the posterior probabilities of the models, calculated by passing the log model evidence through a softmax function. In the case of Fig 8b, we take the model comparison conducted at a prior precision of β^-1^ = 2.25, to justify the model selected in the next section. Fig 8c shows the log model evidence for models 2–6 relative to that of model 1 (the null model), plotted against the prior belief over precision used to generate the design matrix for each mode, and can be thought of as a series of individual model comparisons. The curve for model 3 is in bold to indicate this is the model used for the analysis of participant’s prior beliefs. Note that the null model and models 5–6 do not depend on inferred precision and are therefore invariant to the prior beliefs. These results show that models explicitly containing inferred precision perform better over the range of prior beliefs considered, with the simpler model (model 3) performing best for β^-1^ > 2.25.

Treating model 1 as a null hypothesis, we test the alternate hypotheses to see if they provide a better explanation of the data. The results are shown in Fig 8, with the log model evidence relative to that of the null model (and the R^2^ value to quantify the model fits to the grand mean data) provided for the other models. Model evidence can be read as the probability that a given model would generate the data at hand. The relative (log) model evidence between two models indicates how much better an explanation one model (or hypothesis) is for some given data set compared to another. Crucially, this considers both the accuracy and the complexity of the model, such that larger model evidence indicates that the model either accounts for the data with greater accuracy, or is a simpler explanation with comparable accuracy. This is closely related to other approaches for comparing models, including the Akaike information criterion (AIC) and the Bayesian information criterion (BIC).

Technically, model evidence is the log likelihood (accuracy) minus a (complexity) penalty for the effective number of model parameters. The AIC and BIC may be regarded as approximations to log model evidence, while statistics such as R^2^ reflect accuracy. The key difference between accuracy and log evidence is that log evidence (and its free energy bound) penalises models whose parameters must be moved from their default (prior) values to explain the data (as measured by the Kullback-Leibler divergence between posterior and prior). The AIC and BIC approximate this complexity, but do not take account of whether or not these parameters are used to explain the data. In most model comparison settings, the variational free energy is a better approximation to (log) model evidence than the AIC or BIC [54].

**Fig 9:**
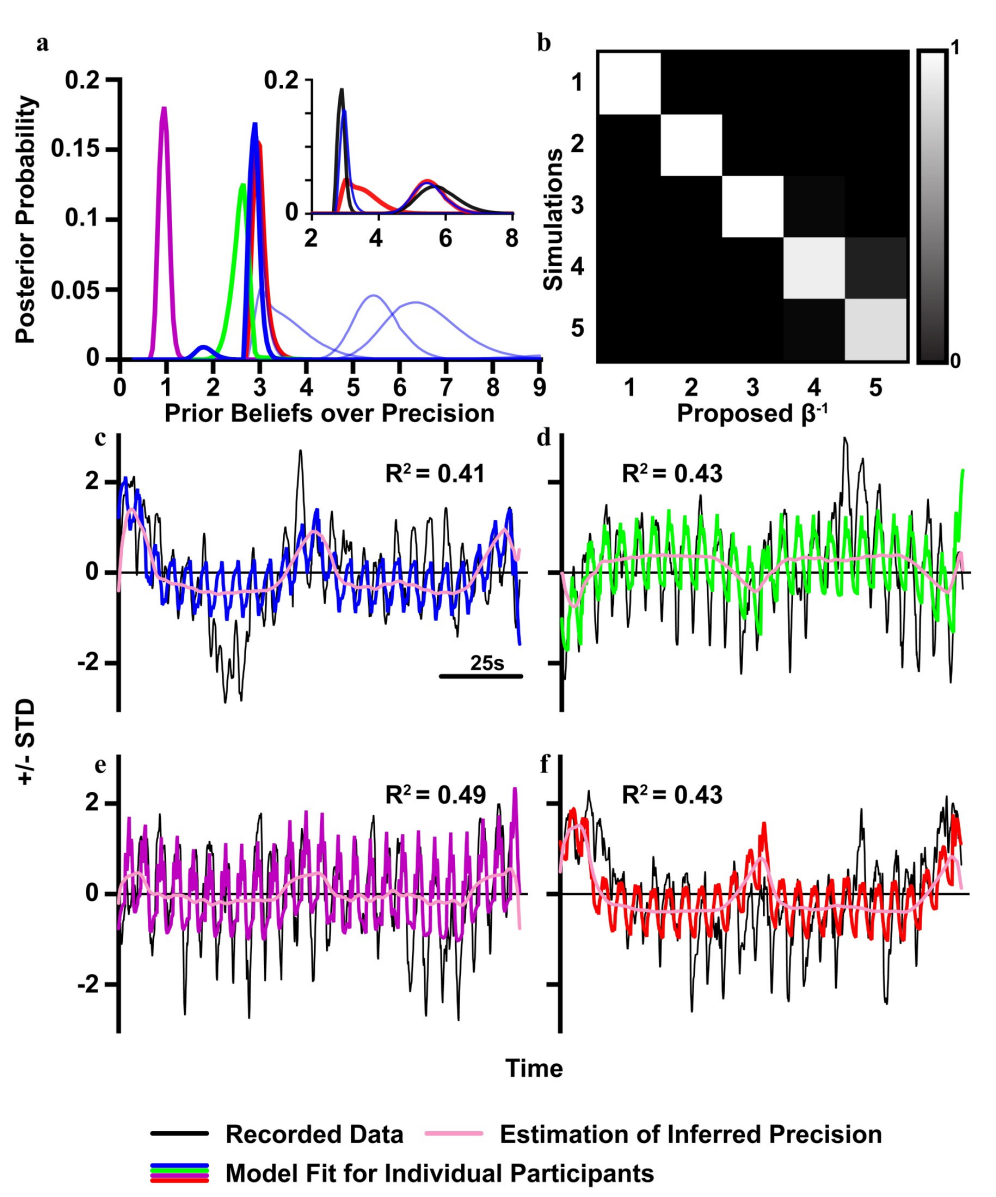
Estimating participants prior beliefs over precision. Using a Bayesian model comparison, we can calculate a posterior distribution over prior beliefs about precision using the evidence for models of observed data, under each level of prior precisions considered. In Fig 9a, posterior distributions for individual participants are shown in blue for 5 of 9, while the remaining 4 are given colours that correspond to their models in parts c-f. The insert for Fig 9a shows the results for two participants when we analyse the data for the different sequences separately, with the red curves indicating the estimated prior precision for the sequences with imprecise A and B matrices, and the black showing the estimated prior precision for sequences with only an imprecise B matrix. The blue lines replicate the data in the main part of Fig 9a, with the two sequences combined. In Fig 9b we show a ‘confusion’ matrix, where the elements show the probability that the prior precision represented by the column was the value used to generate the simulated data represented in each row. Figs 9c-f show the simulated data generated with the most likely prior precision, in the colours given in Fig 9a, overlaid on the recorded data (in black). The estimation of the participant’s inferred precision is also provided in pink, to show the contribution of precision to the model fit, as well as to show the fine scale tracking and responsive of pupil diameter to changes in environmental volatility. The four participants shown had the highest posterior probability in the estimation of their prior beliefs over precision. R^2^ values are given for the model fits for the 4 participants shown.

In the current application of model comparison, we compare models for a range of prior beliefs from 0.3 to 20. Effectively, this is a line search for the optimal prior belief, relative to the null model (which has no dependence on the prior beliefs). Fig 8 shows that at very low prior beliefs, the more complicated model (model 4) is superior, but from a prior precision of β^-1^ > 2.25 the simpler model (model 3) is sufficient. Furthermore, the results confirm that pupillary responses are highly dependent on the β^-1^ (as suggested in Eq 2), and importantly that our model comparison can detect this dependency. With this in mind, we proceeded with a more delicate analysis, using model 3 (since it is superior to the other models over a larger range of precisions, and in particular around the most interesting range of precisions, see Fig 9) to examine the differences among our 9 participants.

### Identification of participant prior beliefs

We may perform the same analysis used above (a Bayesian linear regression with uniform priors) to optimise the model of data generated by each of the 9 participants. This allows one to identify the optimal prior precision for each participant. To do this, following the regression analysis, we can pass the log model evidence of model 3 –for different prior precisions—through a softmax function [53,55] to obtain the posterior probability over prior precision. These results are shown in Fig 9, which shows the posterior probabilities for each of the 9 participants.

In Fig 9, we also report a confusion matrix, constructed by simulating data with different prior precisions (rows), and then computing the posterior probability afforded to each level of prior precision (columns) by each simulated dataset. This is to demonstrate that—if we simulate data—we can easily recover the parameter used to generate the simulations (demonstrating the sensitivity of our measure). To illustrate the face validity of this approach, we simulated data using the parameter values inferred for 4 of the participants. The correspondence between these and the measured data are shown Fig 9c–9f.

Fig 9a shows that our participants displayed a range of β^-1^, with three below the average, four close to the average and two with slightly higher β^-1^. Importantly, we are able to identify an optimal value for all participants. This conclusion is reinforced by Fig 9b, where we show that if we generate simulated time series for 5 similar values of β^-1^, we can accurately assign the precisions to the correct simulation; through model comparison of models with different prior precisions.

This characterisation of model identifiability is reflected in the fact that the highest probabilities lie on the (confusion) matrix diagonal. In other words, we can recover the correct model that generated pupillometry data based on, and only on, the data themselves. To characterise the simulations that are used to find the optimal prior precision, in Fig 9c–9f we overlay the simulations on the recorded data for 4 participants, showing that the regression parameters can be used to generate plausible data. Note in Fig 9c there is a large deviation between the simulation and the recorded data around 400ms; this might explain the double peak seen in the thick blue trace in Fig 9a.

## Discussion

To establish the role of inferred precision (inverse volatility) in explaining pupillometry data, we considered 6 models, each of which has a unique physiological interpretation. Model 1 proposed that optical factors alone are sufficient to explain the data. Had Model 1 won over the others, this would have represented evidence against our hypothesis; namely, that pupil dilation tracks inferred volatility. However, we show that models 2–4—all of which include the inferred precision in some form—are superior to the optical model. Furthermore, the results suggest that the model with inferred precision acting directly on the pupil diameter (Model 3) is the most effective over the largest range of prior precisions; including for the prior precisions shared by most of the participants. Interactions between inferred precision and optical effects have little explanatory value for these data. This suggests that the effect of precision on pupil diameter is distinct and separable from the optical impact (within the bounds of maximum and minimum pupil diameter). Finally, we are able to estimate, in a Bayes optimal fashion, the prior precision for our participants, demonstrating the sensitivity of this estimation in relation to intersubject variability.

While the results presented here are highly complementary to those in previous work taking a Bayesian perspective on pupillary dynamics (most notably by Nassar et al, 2012 and Krishnamurthy et al 2017, 6,31), our approach offers two additional benefits. First, our focus is on inference, as opposed to learning. Intuitively, this generalises previous approaches that focus on the optimisation of parameters of a generative model (learning) to accommodate beliefs about current states of the world (inference), and their changeability. Second, we have formulated our generative model to be consistent with a Markov decision process formulation of Active Inference (16,18). The importance of this is threefold. This sort of model is equipped with a process theory that has been used to account for a range of behavioural and electrophysiological observations, affording it a face validity. In addition to this face validity, the capacity to use exactly the same model to generate pupillary responses and choice behaviour (or evoked EEG responses) provides an opportunity to test the predictive validity of our model. In future work, we hope to be able to use the estimated parameters from pupillary data for individual subjects (or groups of subjects) to predict what one might measure using (for example) electroencephalography. Finally, given established associations between other neurotransmitter systems and parameters of these generative models [2,22,56], we are now in a position to investigate the interactions between these systems (e.g. how does my uncertainty about the changeability of my environment influence my uncertainty in how I am going to act?).

While pupillary dilatation is typically associated with central noradrenergic signalling, it is notable that other neurotransmitter systems have also been correlated with these responses in both humans [57] and animals [58]. As such, the link between pupillary dilatation and the precision of transitions demonstrated here could be a manifestation of other transmitter systems in addition to (or in place of) noradrenaline, as well as different sources of noradrenergic stimulation [59–62]. For these reasons, we can only conclude that neuronal processes upstream of fibres projecting to the pupillary muscles are engaged in estimation of precision (or volatility), and that noradrenaline is the likely substrate of this. However, to implicate noradrenaline with greater confidence, it will be necessary to dissociate this from alternative transmitters. This could be through fMRI, comparing activity in the locus coeruleus, dopaminergic midbrain, and basal forebrain nuclei. Alternatively, it could be through the use of pharmacological intervention, exploring whether a central noradrenergic blockade abolishes the responses observed here. These experiments, when paired with a suitable paradigm to probe changes in likelihood mappings, could be used to further explore the theories of Yu and Dayan and others [2,56,63,64] in probing the neurotransmitter systems that underlie different forms of uncertainty.

Given recent work on the role of aberrant prior beliefs in autism and anxiety disorders, we also suggest that the techniques introduced above could be used to quantify group differences between neurotypical persons and people with autism. In practical terms, this would provide clinicians with a tool to quantitatively phenotype patients and provide a diagnostic aid for autism. Recent findings suggest that these measures may correlate with the severity of symptoms [14]. This suggests there may be utility in this type of phenotyping in quantifying the effects of therapeutic interventions. However, if this was to be used as a diagnostic tool, a change in experimental paradigm would be needed; autism spectrum disorders are often diagnosed very early in life (around 3–4 years old) [65], an age at which children are often not yet able to count. The first step to such a tool would be to use the current paradigm to examine differences between a small group of neurotypical people and patients with autism, within the age range examined in this work (18–35). If these experiments were successful in finding differences between the two sets of participants, subsequent studies could examine the efficacy of the paradigm in younger age groups, adjusting the paradigm to suit those not yet able to count—and those who find it difficult to focus on the stimulus.

Finally, we refer to the introduction and our argument that belief updating over the precision of state transitions is essential for intelligent life. While this work simply shows that humans do appear to use Bayes optimal updating for beliefs regarding volatile state transitions, it provides a solid framework from which to launch further exploration of the subtleties of this precision updating, including its interaction with belief updating for precision of likelihood mappings and for actions. With a solid theoretical and practical understanding of these concepts, the leap to a general artificial intelligence would be less of a jump, and almost a trivial consequence of (variational) optimality principles.

## Supporting information

S1 Data Participant 1. Zip file containing the pupillometry data for participant 1.
(ZIP)

S2 Data Participant 2. Zip file containing the pupillometry data for participant 2.
(ZIP)

S3 Data Participant 3. Zip file containing the pupillometry data for participant 3.
(ZIP)

S4 Data Participant 4. Zip file containing the pupillometry data for participant 4.
(ZIP)

S5 Data Participant 5. Zip file containing the pupillometry data for participant 5.
(ZIP)

S6 Data Participant 6. Zip file containing the pupillometry data for participant 6.
(ZIP)

S7 Data Participant 7. Zip file containing the pupillometry data for participant 7.
(ZIP)

S8 Data Participant 8. Zip file containing the pupillometry data for participant 8.
(ZIP)

S9 Data Participant 9. Zip file containing the pupillometry data for participant 9.
(ZIP)
